# Decreased odds of depressive symptoms and suicidal ideation with higher education, depending on sex and employment status

**DOI:** 10.1371/journal.pone.0299817

**Published:** 2024-04-03

**Authors:** Vanessa K. Tassone, Sophie F. Duffy, Sarah Dunnett, Josheil K. Boparai, Valentina Zuluaga Cuartas, Hyejung Jung, Michelle Wu, Navya Goel, Wendy Lou, Venkat Bhat

**Affiliations:** 1 Interventional Psychiatry Program, St. Michael’s Hospital, Toronto, Ontario, Canada; 2 Department of Biostatistics, Dalla Lana School of Public Health, University of Toronto, Toronto, Ontario, Canada; 3 Institute of Medical Science, Medical Sciences Building, University of Toronto, Toronto, Ontario, Canada; 4 Mental Health and Addictions Services, St. Michael’s Hospital, Toronto, Ontario, Canada; 5 Department of Psychiatry, University of Toronto, Toronto, Ontario, Canada; University of Bern: Universitat Bern, SWITZERLAND

## Abstract

**Background:**

Higher education is associated with reduced depressive symptoms and requires investment without guaranteed employment. It remains unclear how sex and employment status together contribute to the association between mental health and educational attainment. This study investigated the role of sex and employment status together in the associations of 1) depressive symptoms and 2) suicidal ideation with education.

**Methods:**

Using 2005–2018 National Health and Nutrition Examination Survey data, cross-sectional analyses were conducted on individuals ≥20 years who completed the depression questionnaire and reported their employment status and highest level of education. Survey-weighted multivariable logistic regression models were used to explore how depressive symptoms and suicidal ideation are associated with educational attainment in an analysis stratified by sex and employment status. To account for multiple testing, a significance level of *a* < 0.01 was used.

**Results:**

Participants (*n* = 23,669) had a weighted mean age of 43.25 (*SD* = 13.97) years and 47% were female. Employed females (aOR = 0.47, 95% CI 0.32, 0.69), unemployed females (aOR = 0.47, 95% CI 0.29, 0.75), and unemployed males (aOR = 0.31, 95% CI 0.17, 0.56) with college education had reduced odds of depressive symptoms compared to those with high school education. Employed females with college education also had reduced suicidal ideation odds compared to those with high school education (aOR = 0.41, 95% CI 0.22, 0.76).

**Conclusions:**

Females demonstrated significant associations between depressive symptoms and education, regardless of employment status, whereas males demonstrated an association only if unemployed. Employed females, in particular, demonstrated a significant association between suicidal ideation and education. These findings may inform future research investigating the underlying mechanisms and etiology of these sex-employment status differences in the association between mental health and education.

## Introduction

Major depressive disorder (MDD) is a leading contributor to disability worldwide [1], with North America having one of the greatest incidences of MDD globally [2]. The lifetime prevalences of suicidal ideation and attempts in individuals with MDD are 40.3% and 31%, respectively [3, 4]. Females are more likely than males to experience depression and suicidal ideation [5, 6]; however, male suicide mortality is 3 to 4 times that of females [7].

Unemployed adults are known to have higher depression odds than their employed counterparts [8, 9], however, these associations vary by sex. A meta-analysis by Paul and Moser [10] found that the negative effects of unemployment were greater for men than women. Similarly, Andersen et al [11] found a higher prevalence of minor depression in non-employed men than in non-employed women. Suicidal ideation, often a symptom of depression, is suggested to be increased in unemployed males, but not unemployed females [12].

Research suggests that three pathways may explain the association between unemployment and depression. The causal hypothesis suggests that individuals may experience poor mental health as a result of unemployment [13, 14]. Becoming unemployed may lead to a loss of self-worth, personal identity, and perceived sense of control over one’s life, as well as financial instability and a decline in overall well-being [15–17]. Regaining employment may restore mental health to some degree [18]. The selection hypothesis suggests that poor mental health causes unemployment [10, 13, 14]. The causal directions proposed in each of these hypotheses may also operate simultaneously, resulting in bidirectionality between poor mental health and unemployment [10, 19, 20]. The last hypothesis suggests that an external factor, such as educational attainment, may confound the relationship between unemployment and depression [21].

The relationship between education and employment is well established. Individuals with higher education are generally employed at greater rates [22], while those with basic education are more likely to transition out of paid employment due to disability, early retirement, or fulfillment of domestic tasks and care responsibilities [23]. While higher education provides opportunities in the form of employment and higher income [24], it is not without costs. Education is considered a personal investment in human capital, as the opportunity cost of achieving higher education is greater than forgoing it and entering the labor force sooner [18, 25]. Obtaining more years of education to develop the skills needed for a chosen career may also be the product of greater personal investment and identity in one’s career [26]. While higher education generally protects against depressive symptoms and suicidal ideation [27, 28], it is possible that violated expectations for employment following higher education may result in mental discomfort.

Similar to employment, the relationship between depression and education, as well as suicidal ideation and education, is sex-dependent. Although both males and females experience benefits from higher education in reducing depressive symptoms, this relationship is generally stronger for females [29–31]. Additionally, higher education is associated with reduced suicidal ideation among females only [12]. A male-only study found no significant association between education and suicidal ideation, however, there was a negative association with suicide attempt [32]. Sex differences in education may posit interesting mental health trajectories, as females attaining higher education have demonstrated higher degrees of self-help, suggesting improved health literacy and positive impacts on psychosocial skills, compared to their male counterparts [33]. The recent decade has shown changing sex dynamics as higher education has shifted from male- to female-dominated in the United States (US) [5, 22, 30].

As sex and gender roles shift over time, research must reflect the current state of such dynamics. Studies suggest that while males are more adversely affected by unemployment [10–12], females might experience a stronger protective effect of higher education [30]. Therefore, an exploration considering all three variables (i.e., sex, employment status, and education) is warranted to elucidate whether the relationship between mental health and educational attainment for each sex remains consistent for those with differing employment statuses. Given the previously described sex-based associations, it may be expected that:

- Hypothesis 1: The association between education and mental health outcomes will be significant among employed females but not males
  ○ Hypothesis 1a: Employed females with higher education will experience significantly reduced odds of depressive symptoms and suicidal ideation
  ○ Hypothesis 1b: Employed females with less than high school education might experience significantly increased odds of depressive symptoms and suicidal ideation
- Hypothesis 2: The association between education and mental health outcomes will be significant among unemployed females but not males
  ○ Hypothesis 2a: Unemployed females with higher education will experience significantly reduced odds of depressive symptoms and suicidal ideation
  ○ Hypothesis 2b: Unemployed females with less than high school education might experience significantly increased odds of depressive symptoms and suicidal ideation

To the authors’ knowledge, these hypotheses are unexplored in the existing literature. As such, the purpose of this study was to perform a sex and employment status stratified examination of how depressive symptoms and suicidal ideation are associated with educational attainment.

## Methods

### Study population

This study used data from participants who were recruited for the 2005–2018 National Health and Nutrition Examination Survey (NHANES). NHANES contains a series of cross-sectional datasets that are representative of the non-institutionalized resident US population. This survey series is conducted by the National Center for Health Statistics (NCHS), a division of the Centers for Disease Control and Prevention. The multistage probability sampling of participants is conducted by the stepwise selection of counties as the primary sampling unit, then block segments within counties, households within block segments, and finally individuals within households [34–37]. The NHANES protocol was approved by the Research Ethics Review Board of the NCHS and written informed consent was obtained from participants prior to data collection. The authors did not have access to information that could be used to identify participants after data collection.

Participants were excluded from this study if they were not working due to retirement, being a student, taking care of family, or reported “other” as their reason for not working [8]. Participants were also excluded if they were <20 years of age since some of the questions used to collect data for the current analyses were only presented to those who were a minimum of 20 years old. Included participants were required to have responded to items 1–9 on the Mental Health—Depression Screener questionnaire (DPQ), report the type of work that they did in the last week (OCD150) on the Occupation questionnaire (OCQ), and report their highest level of education (DMDEDUC2) from the Demographic Data (DEMO).

### Exposure variables

Educational attainment was categorized using responses to DMDEDUC2 on the DEMO questionnaire. Responses were categorized as <high school, high school, some college/Associate of Arts degree, or college or above. Employment status was dichotomized as employed or unemployed. Based on the responses to OCD150 on the OCQ questionnaire, employed individuals were classified as those who reported working at a job or business or with a job or business but not at work in the last week. Unemployed individuals were classified as those who reported looking for work or were not working at a job or business in the last week. In particular, unemployed individuals included those who were not working due to disability or health-related reasons or were on lay-off in the last week. We opted to characterize unemployment in this way because–unlike retirees, students, or family carers who were not employed but were excluded from the current study–those who were unemployed due to disability, health-related reasons, or lay-off could be perceived as being unwillingly selected into unemployment. While previous work has also included those who were not working due to disability or health-related reasons in analyses [38], other research has limited unemployment to those who were looking for work or were on lay-off [39]. As such, a sensitivity analysis was conducted wherein participants who were not working due to disability or health-related reasons were excluded from the study population since, similar to those who were retired, students, or family carers, these individuals were not in the labour force. Sex was dichotomized as male or female based on the two response options available in NHANES.

### Outcome measures

The primary outcome of this study was depressive symptoms, which was assessed using the nine-item Patient Health Questionnaire (PHQ-9) from the DPQ. The PHQ-9 is a self-report measure corresponding to the *Diagnostic Statistical Manual of Mental Disorders*, *Fourth Edition* diagnostic criteria for MDD [40]. Nine questions, probing the frequencies of symptoms of depression within the last 2 weeks, were scored on a four-point Likert scale from “0” (not experiencing the symptom) to “3” (experiencing the symptom nearly every day). The PHQ-9 has been validated and is a reliable tool for depression diagnosis with high specificity and sensitivity [40–42]. PHQ-9 total scores were dichotomized with scores ≥10 indicating presence of depressive symptoms, and scores <10 indicating absence of depressive symptoms [42].

The secondary outcome of this study was suicidal ideation, assessed using item nine from the PHQ-9. Participants who responded to this item with the answer “not at all” were classified as having no suicidal ideation. Responses of “several days,” “more than half the days,” or “nearly every day” were classified as experiencing suicidal ideation. Item nine on the PHQ-9 has been shown to have a high negative predictive value and specificity, as well as a moderate sensitivity. It is recommended as a screening tool for suicidal ideation due to a low positive predictive value [43, 44].

### Covariates

Covariates were selected based on existing knowledge from the literature to adjust for potential confounding bias [30]. Covariates included age (continuous by 1-year increases) [45], race (non-Hispanic white, non-Hispanic black, Hispanic, other/multi-racial), marital status (married/living with partner or not) [46], and NHANES survey cycle (2-year intervals).

### Statistical analysis

Data analysis was performed using Mobile Examination Center (MEC) survey weights in R v 4.2.2 with the package “survey.” MEC survey weights were used to account for the clustered sample design, oversampling, survey non-response, and post-stratification adjustments so that the results could be generalized to the US population. Survey weights were divided by seven to account for combining seven survey cycles to make up the study population. The study population was divided into four groups based on both employment status and sex. To adjust for multiple testing and reduce the risk of type I error, significance was established at *P* < 0.01 using two-tailed hypothesis testing. Significant demographic differences between any of these four groups were determined using an analysis of variance for continuous variables and a χ^2^ test for categorical variables. An analysis examining the reasons for unemployment was conducted between sexes and proportions were tested using a χ^2^ test for categorical variables. Multivariable logistic regression models were used to estimate the association between education and depressive symptoms, as well as suicidal ideation, adjusting for age, race, marital status, and NHANES survey cycle among each of the four groups. Participants were excluded from analysis if they had missing data or responded “refused” or “I don’t know” to variables of interest.

## Results

### Demographic characteristics

**Fig 1:**
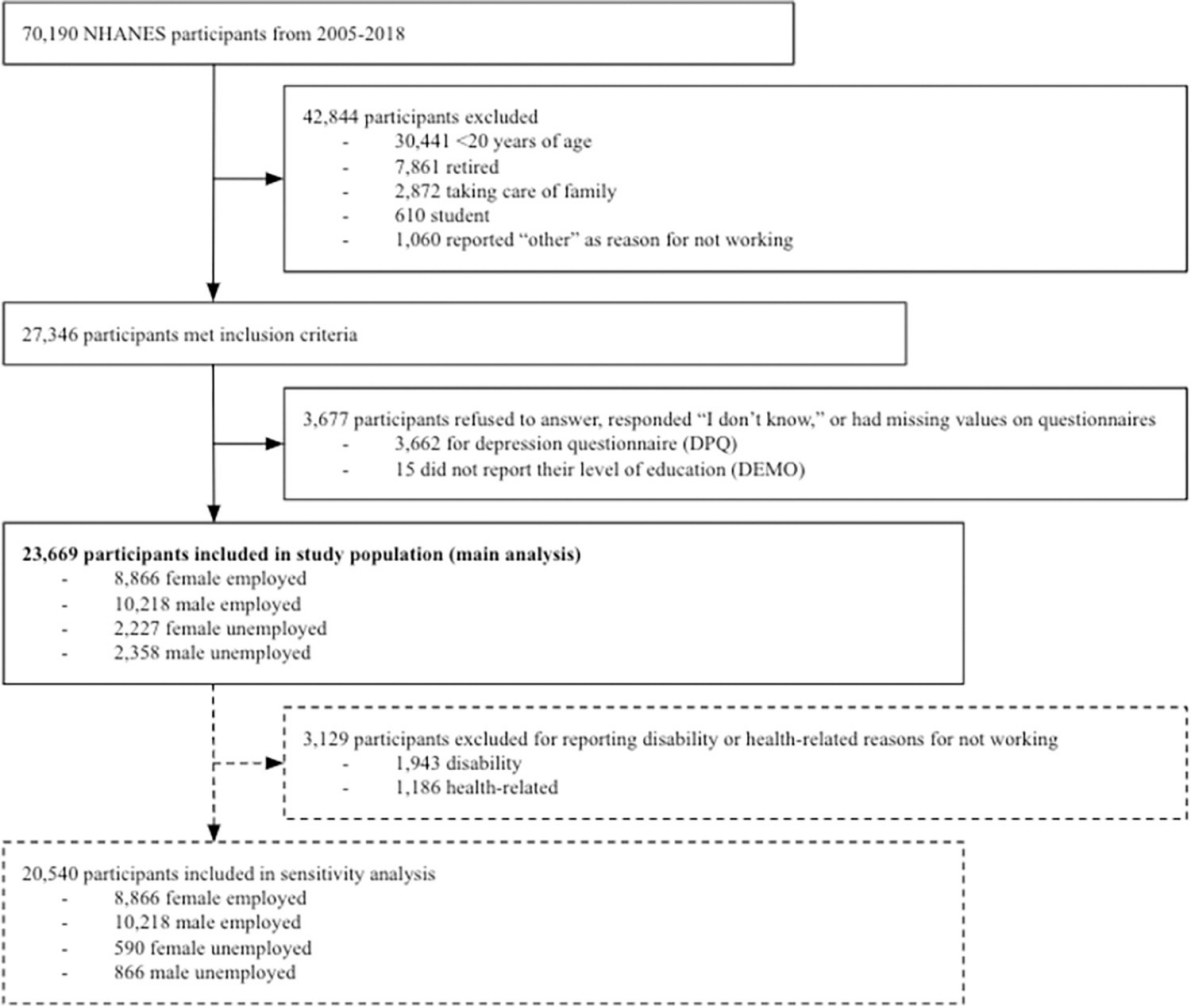
Flow chart of participant inclusion from NHANES 2005–2018 surveys.

A total of 23,669 individuals aged 20 to 85 years were included in this study (Fig 1). The weighted prevalence of depressive symptoms and suicidal ideation was 7.96% and 3.29%, respectively. Unemployed females had the highest prevalence of depressive symptoms and suicidal ideation, followed by unemployed males, employed females, and employed males. Employed females pursued education beyond high school at the highest rate, followed by employed males, unemployed females, and unemployed males. Table 1 and S1 Table present the demographic characteristics for the study population and the sensitivity analysis, respectively.

**Table 1:** Demographic characteristics of the NHANES 2005–2018 study population (*n* = 23,669), stratified by sex and employment status.

	Female Employed	Male Employed	Female Unemployed	Male Unemployed	*P* value
*n*	8,866	10,218	2,227	2,358	
**Depressive symptoms** = Yes (%)	632 (6.58)	377 (3.63)	709 (31.45)	475 (19.78)	<0.001
**Suicidal ideation** = Yes (%)	230 (2.35)	217 (1.98)	243 (10.20)	224 (10.01)	<0.001
**Age**, mean (SD)	42.72 (13.65)	42.34 (13.66)	48.92 (14.81)	46.28 (15.03)	<0.001
**Race** (%)					<0.001
Non-Hispanic White	3,546 (67.40)	4,134 (67.20)	881 (61.28)	955 (59.64)	
Hispanic	2,252 (12.90)	2,855 (16.16)	527 (13.44)	522 (14.86)	
Non-Hispanic Black	2,043 (12.10)	1,991 (9.13)	652 (18.87)	693 (18.18)	
Other / Multi-racial	1,025 (7.59)	1,238 (7.50)	167 (6.41)	188 (7.33)	
**Marital status** = Not Married / Living with partner (%)	3,834 (38.57)	3,131 (30.14)	1,353 (55.37)	1,223 (53.08)	<0.001
**Education** (%)					<0.001
< High school	1,342 (9.47)	2,208 (13.96)	755 (25.93)	870 (28.41)	
High school	1,783 (19.48)	2,395 (23.85)	537 (26.12)	691 (30.87)	
Some college / Associate of Arts degree	3,146 (35.48)	2,874 (29.95)	691 (32.53)	585 (28.33)	
College or above	2,595 (35.56)	2,741 (32.23)	244 (15.42)	212 (12.38)	

Continuous characteristics reported with weighted mean and standard deviation while categorical characteristics reported with unweighted frequency and weighted percentage. *P* values reported using survey weights.

A higher proportion of females than males were unemployed (15.59% vs. 13.28%). After exploring the reported reasons for unemployment by sex, a greater proportion of females than males were unemployed due to health reasons and disability, while a greater proportion of males than females were unemployed due to lay-off or were looking for work (Table 2). S2 Table presents the reasons for unemployment for those included in the sensitivity analysis.

**Table 2:** Reason for unemployment among those unemployed in the study population, stratified by sex.

	Female Unemployed	Male Unemployed	*P* value
***n* (%)**	2,227 (15.59)	2,358 (13.28)	
**Reason for Unemployment**			<0.001
Health-related (%)	677 (28.75)	509 (20.10)	
Disability (%)	960 (40.81)	983 (39.45)	
Lay-off (%)	162 (7.67)	228 (10.10)	
Looking for work (%)	428 (22.76)	638 (30.36)	

Results reported using unweighted frequency and weighted percentage. *P* values reported using survey weights.

### Depressive symptoms and educational attainment

In both the unadjusted (S3 Table) and adjusted (Table 3) models, employed females with less than high school education had significantly increased odds of depressive symptoms, compared to those with high school education. Additionally, all groups with a college degree, except employed males, had significantly reduced odds of depressive symptoms compared those with high school education. The associations between depressive symptoms and education became non-significant for unemployed females and males in the sensitivity analysis (S4 and S5 Tables).

**Table 3:** Adjusted logistic regression of depressive symptoms and educational attainment, stratified by sex and employment status (study population).

	Female Employed		Male Employed		Female Unemployed		Male Unemployed	
	aOR (95% CI)	P value	aOR (95% CI)	P value	aOR (95% CI)	P value	aOR (95% CI)	P value
Education								
High school	1 (Referent)		1 (Referent)		1 (Referent)		1 (Referent)	
< High school	1.75 (1.22, 2.50)	0.003*	1.46 (1.01, 2.11)	0.050	1.06 (0.76, 1.46)	0.74	1.20 (0.85, 1.67)	0.30
Some college / Associate of Arts degree	1.03 (0.76, 1.41)	0.84	1.23 (0.88, 1.70)	0.23	0.84 (0.63, 1.13)	0.26	0.76 (0.53, 1.09)	0.13
College or above	0.47 (0.32, 0.69)	<0.001*	0.66 (0.43, 1.02)	0.07	0.47 (0.29, 0.75)	0.002*	0.31 (0.17, 0.56)	<0.001*

* indicates statistical significance (P < 0.01). aOR = adjusted odds ratio. CI = confidence interval. Covariates include age, race, marital status, and NHANES survey cycle.

### Suicidal ideation and educational attainment

In both the unadjusted (S6 Table) and adjusted (Table 4) models, employed females with college or above education had significantly lower odds of suicidal ideation when compared to those with high school education. The sensitivity analysis demonstrated similar results (S7 and S8 Tables).

**Table 4:** Adjusted logistic regression of suicidal ideation and educational attainment, stratified by sex and employment status (study population).

	Female Employed		Male Employed		Female Unemployed		Male Unemployed	
	aOR (95% CI)	P value	aOR (95% CI)	P value	aOR (95% CI)	P value	aOR (95% CI)	P value
Education								
High school	1 (Referent)		1 (Referent)		1 (Referent)		1 (Referent)	
< High school	1.96 (1.12, 3.43)	0.02	1.11 (0.70, 1.77)	0.66	0.92 (0.62, 1.38)	0.70	1.03 (0.65, 1.65)	0.89
Some college / Associate of Arts degree	0.74 (0.45, 1.22)	0.24	1.10 (0.71, 1.70)	0.66	0.77 (0.50, 1.18)	0.23	1.01 (0.60, 1.72)	0.96
College or above	0.41 (0.22, 0.76)	0.006*	0.62 (0.35, 1.09)	0.10	0.81 (0.43, 1.52)	0.51	0.52 (0.23, 1.17)	0.117

* indicates statistical significance (P < 0.01). aOR = adjusted odds ratio. CI = confidence interval. Covariates include age, race, marital status, and NHANES survey cycle.

## Discussion

This study examined the association between depressive symptoms and educational attainment, as well as the association between suicidal ideation and educational attainment, using a nationally representative sample of the US population, stratified by sex and employment status. Our results revealed that employed females with less than high school education demonstrated increased odds of depressive symptoms compared to those with high school education. Females with college or above education had decreased odds of depressive symptoms, regardless of employment status. Males also demonstrated decreased odds of depressive symptoms with college or above education, however, this was only significant amongst the unemployed. The associations between depressive symptoms and education became non-significant in unemployed participants, regardless of sex, when those who were not working due to disability or health-related reasons were excluded from analyses. Employed females were the only group to demonstrate a significant association between suicidal ideation and education, such that those with college or above education demonstrated decreased odds of this specific depressive symptom.

The results of this study supported our hypothesis that employed females with higher education would have a significant reduction in depressive symptom odds, whereas employed males would not. The results also supported our hypothesis that employed females with less than high school education would experience significantly increased depressive symptom odds. Higher education may provide improved health literacy and help-seeking behaviors, resulting in positive implications on mental health. For instance, Warner et al [33] found that women had significantly higher self-help scores with increased educational attainment, while men did not. Higher education also appears to enhance one’s sense of control, thus yielding improvement in mental well-being, particularly among women, as they apply the skills learned through formal education to their lives [30]. While education has positive effects on reducing depressive symptoms in females, wealth has been found to be a protective socioeconomic factor against depression for males [29]. These differential sex-based relationships may have historical roots in the perception of stability in employment, which may contribute to reducing depressive outcomes. Historically, males were the sole breadwinners in heterosexual households, making their level of income deterministic of the household’s wealth. This notion is challenged in present-day US households which follow a dual-breadwinner model as egalitarian views on gender roles are becoming more prevalent and traditionalist views are declining [47, 48]. Despite these changes, females continue to receive unequal pay for equal work compared to their male counterparts [49]. Thus, it is possible that females find less security in wealth and more security in higher education that can provide them with the stability and mental wellness that often accompanies having greater employment opportunities and income. Notably, the male-female wage gap is neutralizing over time, possibly leading females to also seek greater security in wealth [49]. Further research on the sex- and gender-based differences in net wealth and depressive symptoms is needed, as well as the etiology of the sex differences in the depressive symptom and education relationship.

The current study demonstrated that both unemployed females and males had reduced depressive symptom odds with higher education when the total study population was considered. This was contrary to our hypothesis that an association between mental health and education would exist among unemployed females only, thus demonstrating the importance of considering sex and employment status together when assessing this relationship. As the required qualifications for employment increase overtime, unemployed individuals with higher education might find security in their favourable employment prospects [50, 51], regardless of sex, which could be protective against depressive symptoms. However, the association between depressive symptoms and education became non-significant in both sexes after removal of those who were not working due to disability or health-related reasons from analyses. This difference in findings might be explained by an early theory regarding causal attributions of unemployment [52, 53]. Feather and Davenport [52, 53] reported that depressed affect was positively associated with causal attributions which emphasized stable and frustrating external factors, such as social, economic, and political forces, as reasons for unemployment (e.g., in people who were needing or looking for work). In contrast, individuals who reported internal attributions for unemployment appeared to exhibit less negative affect [53], which was supported by subsequent research reporting that an internal locus of control was associated with lower levels of depression [54]. As such, grouping individuals who might attribute unemployment to internal factors, such as health-related reasons or disability, with those who were looking for work or were on lay-off might have skewed the association between depressive symptoms and education in the current study, regardless of sex. It is worth noting, however, that the unemployment group drastically decreased in size upon removing individuals who were not working due to disability or health-related reasons from analyses, which could have also impacted the results of the sensitivity analysis.

The odds of suicidal ideation, a possible symptom of major depression [55], were significantly decreased amongst employed females with college or above education. This is in line with a previous study which demonstrated a female-specific protective effect of higher education against suicidal thoughts [12]. The literature on the association between suicidal ideation and education shows mixed results. Studies demonstrate that, in addition to sex, age may be an important moderator. Research has shown that young adults with a college degree (2- or 4-year degree) had reduced odds of suicidal ideation, while middle-aged adults with a 4-year college degree had higher odds of suicidal ideation [45]. A study specifically in young adults found that among those with more than high school education, suicidal ideation was not associated with unemployment within the last two years, however, it was positively associated with lifetime unemployment [56]. Within the current study, age was only considered as a covariate in analyses and participants were categorized as unemployed based on whether they worked within the last week, both of which could potentially explain the lack of association between suicidal ideation and education in unemployed females. Future studies may analyze the associations investigated here with an added interest for the effects of age and duration of unemployment.

### Limitations

Due to the cross-sectional nature of NHANES, causation could not be established in this study. The questionnaires used by NHANES are also limited by their self-reported nature, subjecting responses to potential recall, social desirability, and non-response biases. An additional limitation of the NHANES dataset includes responses to the sex question being restricted to “male” or “female”, without consideration of those who are intersex. As such, potential intersex individuals who felt compelled to respond to the sex question would have been inaccurately dichotomized as male or female. Detection of suicidal ideation may also require an improved method in place of item nine on the PHQ-9 due to evidence suggesting that it has limited utility in certain demographic and clinical groups [57]. However, a systematic review suggests that there is no gold standard instrument to achieve this and a new tool is needed [58]. It is possible that external events, such as the Great Recession, influenced the results of our analyses. Due to constraints of the NHANES dataset, we were limited in our ability to account for such external factors (i.e., by adjusting for survey cycle). Similarly, due to restrictions of the NHANES dataset, we were unable to account for the gender pay-gap in analyses. Finally, NHANES asks participants to report on their work situation within the last week, making it impossible to account for the total length of time that participants were not working for in analyses. As such, the “unemployment” group may be characterized by high heterogeneity not only in terms of length of unemployment, but also in terms of the number of times that one has been unemployed, which could confound the results of the current study.

## Conclusion and future directions

This study adds to the existing literature by further exploring the sex-based associations between depressive symptoms and education, as well as between suicidal ideation and education, from the perspective of employment status. Specifically, employed females with less than high school education demonstrated increased odds of depressive symptoms. Employed and unemployed females, as well as unemployed males, had reduced depressive symptom odds with higher education. Employed females with higher education also demonstrated decreased odds of suicidal ideation. These findings suggest that there are interesting differences in depressive symptom and suicidal ideation odds with higher education based on sex and employment status. They also highlight potential vulnerability amongst employed females with less than high school education who might benefit from efforts to promote secondary school completion, including early intervention at the school-age or provision of incentives to encourage re-entry into the educational system at the adult-age, and preventative mental health programming to promote resiliency.

Future studies should further investigate how higher education and employment status may together influence sex-based dynamics in depressive symptoms and suicidal ideation while also accounting for health status amongst the unemployed (i.e., not working due to health-related reasons or disability vs. looking for work or on lay-off), length of unemployment, the type of job one would be working if they were not unemployed (i.e., blue- or white-collar work), net wealth, ethnicity, and the gender pay-gap. Longitudinal studies may aid in understanding the directionality of this relationship.

## Supporting information

S1 Table Demographic characteristics of the sensitivity analysis (i.e., individuals not working due to disability or health-related reasons excluded; n = 20,540), stratified by sex and employment status. Continuous characteristics reported with weighted mean and standard deviation while categorical characteristics reported with unweighted frequency and weighted percentage. P values reported using survey weights.
(DOCX)

S2 Table Reason for unemployment among those unemployed in the sensitivity analysis (i.e., individuals not working due to disability or health-related reasons excluded), stratified by sex. Results reported using unweighted frequency and weighted percentage. P values reported using survey weights.
(DOCX)

S3 Table Unadjusted logistic regression of depressive symptoms and educational attainment, stratified by sex and employment status (study population). * indicates statistical significance (P < 0.01). OR = odds ratio. CI = confidence interval.
(DOCX)

S4 Table Unadjusted logistic regression of depressive symptoms and educational attainment, stratified by sex and employment status (sensitivity analysis). * indicates statistical significance (P < 0.01). OR = odds ratio. CI = confidence interval.
(DOCX)

S5 Table Adjusted logistic regression of depressive symptoms and educational attainment, stratified by sex and employment status (sensitivity analysis). * indicates statistical significance (P < 0.01). aOR = adjusted odds ratio. CI = confidence interval. Covariates include age, race, marital status, and NHANES survey cycle.
(DOCX)

S6 Table Unadjusted logistic regression of suicidal ideation and educational attainment, stratified by sex and employment status (study population). * indicates statistical significance (P < 0.01). OR = odds ratio. CI = confidence interval.
(DOCX)

S7 Table Unadjusted logistic regression of suicidal ideation and educational attainment, stratified by sex and employment status (sensitivity analysis). * indicates statistical significance (P < 0.01). OR = odds ratio. CI = confidence interval.
(DOCX)

S8 Table Adjusted logistic regression of suicidal ideation and educational attainment, stratified by sex and employment status (sensitivity analysis). * indicates statistical significance (P < 0.01). aOR = adjusted odds ratio. CI = confidence interval. Covariates include age, race, marital status, and NHANES survey cycle.
(DOCX)
