# Supplementary material for: Decreased odds of depressive symptoms and suicidal ideation with higher education, depending on sex and employment status
Source: PLoS One. 2024 Apr 3;19(4):e0299817. doi: 10.1371/journal.pone.0299817 (PMC10990184; doi:10.1371/journal.pone.0299817)
Supplement: S1 Table — Continuous characteristics reported with weighted mean and standard deviation while categorical characteristics reported with unweighted frequency and weighted percentage. P values reported using survey weights. (DOCX) [file pone.0299817.s001.docx]

**S1 Table. Demographic characteristics of the sensitivity analysis (i.e., individuals not working due to disability or health-related reasons excluded; *n* = 20,540), stratified by sex and employment status.**

|  | **Female Employed** | **Male**  **Employed** | **Female Unemployed** | **Male Unemployed** | *P* value |
| --- | --- | --- | --- | --- | --- |
| *n* | 8,866 | 10,218 | 590 | 866 |  |
| **Depressive symptoms** = Yes (%) | 632 (6.58) | 377 (3.63) | 85 (13.86) | 88 (9.31) | <0.001 |
| **Suicidal ideation** = Yes (%) | 230 (2.35) | 217 (1.98) | 33 (4.65) | 53 (6.14) | <0.001 |
| **Age**, mean (SD) | 42.72 (13.65) | 42.34 (13.66) | 39.62 (14.50) | 37.69 (13.82) | <0.001 |
| **Race** (%) |  |  |  |  | <0.001 |
| Non-Hispanic White | 3,546 (67.40) | 4,134 (67.20) | 197 (55.62) | 306 (52.87) |  |
| Hispanic | 2,252 (12.90) | 2,855 (16.16) | 143 (16.39) | 199 (17.68) |  |
| Non-Hispanic Black | 2,043 (12.10) | 1,991 (9.13) | 186 (20.01) | 267 (20.73) |  |
| Other / Multi-racial | 1,025 (7.59) | 1,238 (7.50) | 64 (7.98) | 94 (8.72) |  |
| **Marital** **status** = Not Married / Living with partner (%) | 3,834 (38.57) | 3,131 (30.14) | 334 (51.47) | 468 (56.30) | <0.001 |
| **Education** (%) |  |  |  |  | <0.001 |
| < High school | 1,342 (9.47) | 2,208 (13.96) | 119 (14.53) | 246 (22.54) |  |
| High school | 1,783 (19.48) | 2,395 (23.85) | 138 (23.35) | 249 (28.01) |  |
| Some college / Associate of Arts degree | 3,146 (35.48) | 2,874 (29.95) | 210 (33.79) | 244 (30.56) |  |
| College or above | 2,595 (35.56) | 2,741 (32.23) | 123 (28.34) | 127 (18.89) |  |

Note. Continuous characteristics reported with weighted mean and standard deviation while categorical characteristics reported with unweighted frequency and weighted percentage. *P* values reported using survey weights.
