# Supplementary material for: Decreased odds of depressive symptoms and suicidal ideation with higher education, depending on sex and employment status
Source: PLoS One. 2024 Apr 3;19(4):e0299817. doi: 10.1371/journal.pone.0299817 (PMC10990184; doi:10.1371/journal.pone.0299817)
Supplement: S2 Table — Results reported using unweighted frequency and weighted percentage. P values reported using survey weights. (DOCX) [file pone.0299817.s002.docx]

**S2 Table. Reason for unemployment among those unemployed in the sensitivity analysis (i.e., individuals not working due to disability or health-related reasons excluded), stratified by sex.**

|  | **Female**  **Unemployed** | **Male**  **Unemployed** | *P* value |
| --- | --- | --- | --- |
| **n (%)** | 590 (5.32) | 866 (5.83) |  |
| **Reason for Unemployment** |  |  | 0.942 |
| Lay-off (%) | 162 (25.20) | 228 (24.96) |  |
| Looking for work (%) | 428 (74.80) | 638 (75.04) |  |

Note. Results reported using unweighted frequency and weighted percentage. *P* values reported using survey weights.
