# Supplementary material for: Decreased odds of depressive symptoms and suicidal ideation with higher education, depending on sex and employment status
Source: PLoS One. 2024 Apr 3;19(4):e0299817. doi: 10.1371/journal.pone.0299817 (PMC10990184; doi:10.1371/journal.pone.0299817)
Supplement: S3 Table — * indicates statistical significance (P < 0.01). OR = odds ratio. CI = confidence interval. (DOCX) [file pone.0299817.s003.docx]

**S3 Table. Unadjusted logistic regression of depressive symptoms and educational attainment, stratified by sex and employment status (study population).**

|  | **Female Employed** | | **Male Employed** | | **Female Unemployed** | | **Male Unemployed** | |
| --- | --- | --- | --- | --- | --- | --- | --- | --- |
|  | OR (95% CI) | *P* value | OR (95% CI) | *P* value | OR (95% CI) | *P* value | OR (95% CI) | *P* value |
| **Education** |  |  |  |  |  |  |  |  |
| High school | 1 (Referent) |  | 1 (Referent) |  | 1 (Referent) |  | 1 (Referent) |  |
| < High school | 1.65 (1.17, 2.34) | 0.006* | 1.24 (0.86, 1.78) | 0.25 | 1.04 (0.77, 1.41) | 0.79 | 1.20 (0.86, 1.67) | 0.30 |
| Some college / Associate of Arts degree | 1.08 (0.80, 1.47) | 0.62 | 1.21 (0.87, 1.67) | 0.26 | 0.86 (0.64, 1.15) | 0.30 | 0.77 (0.54, 1.10) | 0.16 |
| College or above | 0.46 (0.32, 0.67) | <0.001* | 0.62 (0.41, 0.95) | 0.03 | 0.49 (0.31, 0.78) | 0.003* | 0.31 (0.18, 0.57) | <0.001* |

Note. * indicates statistical significance (*P* < 0.01). OR = odds ratio. CI = confidence interval.
