# Supplementary material for: Decreased odds of depressive symptoms and suicidal ideation with higher education, depending on sex and employment status
Source: PLoS One. 2024 Apr 3;19(4):e0299817. doi: 10.1371/journal.pone.0299817 (PMC10990184; doi:10.1371/journal.pone.0299817)
Supplement: S4 Table — * indicates statistical significance (P < 0.01). OR = odds ratio. CI = confidence interval. (DOCX) [file pone.0299817.s004.docx]

**S4 Table. Unadjusted logistic regression of depressive symptoms and educational attainment, stratified by sex and employment status (sensitivity analysis).**

|  | **Female Employed** | | **Male Employed** | | **Female Unemployed** | | **Male Unemployed** | |
| --- | --- | --- | --- | --- | --- | --- | --- | --- |
|  | OR (95% CI) | *P* value | OR (95% CI) | *P* value | OR (95% CI) | *P* value | OR (95% CI) | *P* value |
| **Education** |  |  |  |  |  |  |  |  |
| High school | 1 (Referent) |  | 1 (Referent) |  | 1 (Referent) |  | 1 (Referent) |  |
| < High school | 1.65 (1.17, 2.34) | 0.006* | 1.24 (0.86, 1.78) | 0.25 | 0.70 (0.32, 1.54) | 0.38 | 1.05 (0.57, 1.96) | 0.87 |
| Some college / Associate of Arts degree | 1.08 (0.80, 1.47) | 0.62 | 1.21 (0.87, 1.67) | 0.26 | 1.12 (0.52, 2.39) | 0.77 | 0.69 (0.33, 1.41) | 0.31 |
| College or above | 0.46 (0.32, 0.67) | <0.001* | 0.62 (0.41, 0.95) | 0.03 | 0.41 (0.16, 1.06) | 0.07 | 0.40 (0.16, 1.00) | 0.05 |

Note. * indicates statistical significance (*P* < 0.01). OR = odds ratio. CI = confidence interval.
