# Supplementary material for: Decreased odds of depressive symptoms and suicidal ideation with higher education, depending on sex and employment status
Source: PLoS One. 2024 Apr 3;19(4):e0299817. doi: 10.1371/journal.pone.0299817 (PMC10990184; doi:10.1371/journal.pone.0299817)
Supplement: S5 Table — * indicates statistical significance (P < 0.01). aOR = adjusted odds ratio. CI = confidence interval. Covariates include age, race, marital status, and NHANES survey cycle. (DOCX) [file pone.0299817.s005.docx]

**S5 Table. Adjusted logistic regression of depressive symptoms and educational attainment, stratified by sex and employment status (sensitivity analysis).**

|  | **Female Employed** | | **Male Employed** | | **Female Unemployed** | | **Male Unemployed** | |
| --- | --- | --- | --- | --- | --- | --- | --- | --- |
|  | aOR (95% CI) | *P* value | aOR (95% CI) | *P* value | aOR (95% CI) | *P* value | aOR (95% CI) | *P* value |
| **Education** |  |  |  |  |  |  |  |  |
| High school | 1 (Referent) |  | 1 (Referent) |  | 1 (Referent) |  | 1 (Referent) |  |
| < High school | 1.75 (1.22, 2.50) | 0.003* | 1.46 (1.01, 2.11) | 0.05 | 0.72 (0.31, 1.67) | 0.45 | 1.08 (0.60, 1.93) | 0.81 |
| Some college / Associate of Arts degree | 1.03 (0.76, 1.41) | 0.84 | 1.23 (0.88, 1.70) | 0.23 | 1.07 (0.50, 2.26) | 0.87 | 0.68 (0.34, 1.38) | 0.29 |
| College or above | 0.47 (0.32, 0.69) | <0.001* | 0.66 (0.43, 1.02) | 0.07 | 0.43 (0.18, 1.05) | 0.07 | 0.38 (0.15, 0.98) | 0.05 |

Note. * indicates statistical significance (*P* < 0.01). aOR = adjusted odds ratio. CI = confidence interval. Covariates include age, race, marital status, and NHANES survey cycle.
