# Supplementary material for: Decreased odds of depressive symptoms and suicidal ideation with higher education, depending on sex and employment status
Source: PLoS One. 2024 Apr 3;19(4):e0299817. doi: 10.1371/journal.pone.0299817 (PMC10990184; doi:10.1371/journal.pone.0299817)
Supplement: S6 Table — * indicates statistical significance (P < 0.01). OR = odds ratio. CI = confidence interval. (DOCX) [file pone.0299817.s006.docx]

**S6 Table. Unadjusted logistic regression of suicidal ideation and educational attainment, stratified by sex and employment status (study population).**

|  | **Female Employed** | | **Male Employed** | | **Female Unemployed** | | **Male Unemployed** | |
| --- | --- | --- | --- | --- | --- | --- | --- | --- |
|  | OR (95% CI) | *P* value | OR (95% CI) | *P* value | OR (95% CI) | *P* value | OR (95% CI) | *P* value |
| **Education** |  |  |  |  |  |  |  |  |
| High school | 1 (Referent) |  | 1 (Referent) |  | 1 (Referent) |  | 1 (Referent) |  |
| < High school | 1.95 (1.13, 3.36) | 0.02 | 1.16 (0.73, 1.83) | 0.54 | 1.06 (0.72, 1.57) | 0.77 | 0.99 (0.63, 1.56) | 0.96 |
| Some college / Associate of Arts degree | 0.80 (0.48, 1.34) | 0.41 | 1.07 (0.70, 1.65) | 0.76 | 0.77 (0.50, 1.19) | 0.25 | 1.01 (0.61, 1.68) | 0.96 |
| College or above | 0.41 (0.22, 0.77) | 0.006* | 0.57 (0.33, 0.98) | 0.05 | 0.77 (0.42, 1.42) | 0.41 | 0.47 (0.21, 1.03) | 0.06 |

Note. * indicates statistical significance (*P* < 0.01). OR = odds ratio. CI = confidence interval.
