# Supplementary material for: Decreased odds of depressive symptoms and suicidal ideation with higher education, depending on sex and employment status
Source: PLoS One. 2024 Apr 3;19(4):e0299817. doi: 10.1371/journal.pone.0299817 (PMC10990184; doi:10.1371/journal.pone.0299817)
Supplement: S7 Table — * indicates statistical significance (P < 0.01). OR = odds ratio. CI = confidence interval. (DOCX) [file pone.0299817.s007.docx]

**S7 Table. Unadjusted logistic regression of suicidal ideation and educational attainment, stratified by sex and employment status (sensitivity analysis).**

|  | **Female Employed** | | **Male Employed** | | **Female Unemployed** | | **Male Unemployed** | |
| --- | --- | --- | --- | --- | --- | --- | --- | --- |
|  | OR (95% CI) | *P* value | OR (95% CI) | *P* value | OR (95% CI) | *P* value | OR (95% CI) | *P* value |
| **Education** |  |  |  |  |  |  |  |  |
| High school | 1 (Referent) |  | 1 (Referent) |  | 1 (Referent) |  | 1 (Referent) |  |
| < High school | 1.95 (1.13, 3.36) | 0.02 | 1.16 (0.73, 1.83) | 0.54 | 0.79 (0.28, 2.21) | 0.65 | 1.12 (0.46, 2.76) | 0.80 |
| Some college / Associate of Arts degree | 0.80 (0.48, 1.34) | 0.41 | 1.07 (0.70, 1.65) | 0.76 | 0.79 (0.30, 2.10) | 0.64 | 0.81 (0.33, 1.99) | 0.65 |
| College or above | 0.41 (0.22, 0.77) | 0.006* | 0.57 (0.33, 0.98) | 0.05 | 0.52 (0.14, 1.90) | 0.32 | 0.71 (0.19, 2.62) | 0.61 |

Note. * indicates statistical significance (*P* < 0.01). OR = odds ratio. CI = confidence interval.
