# Supplementary material for: Decreased odds of depressive symptoms and suicidal ideation with higher education, depending on sex and employment status
Source: PLoS One. 2024 Apr 3;19(4):e0299817. doi: 10.1371/journal.pone.0299817 (PMC10990184; doi:10.1371/journal.pone.0299817)
Supplement: S8 Table — * indicates statistical significance (P < 0.01). aOR = adjusted odds ratio. CI = confidence interval. Covariates include age, race, marital status, and NHANES survey cycle. (DOCX) [file pone.0299817.s008.docx]

**S8 Table. Adjusted logistic regression of suicidal ideation and educational attainment, stratified by sex and employment status (sensitivity analysis).**

|  | **Female Employed** | | **Male Employed** | | **Female Unemployed** | | **Male Unemployed** | |
| --- | --- | --- | --- | --- | --- | --- | --- | --- |
|  | aOR (95% CI) | *P* value | aOR (95% CI) | *P* value | aOR (95% CI) | *P* value | aOR (95% CI) | *P* value |
| **Education** |  |  |  |  |  |  |  |  |
| High school | 1 (Referent) |  | 1 (Referent) |  | 1 (Referent) |  | 1 (Referent) |  |
| < High school | 1.96 (1.12, 3.43) | 0.02 | 1.11 (0.70, 1.77) | 0.66 | 0.62 (0.21, 1.86) | 0.39 | 1.39 (0.57, 3.42) | 0.47 |
| Some college / Associate of Arts degree | 0.74 (0.45, 1.22) | 0.24 | 1.10 (0.71, 1.70) | 0.66 | 0.63 (0.23, 1.73) | 0.38 | 0.96 (0.38, 2.44) | 0.93 |
| College or above | 0.41 (0.22, 0.76) | 0.006* | 0.62 (0.35, 1.09) | 0.10 | 0.45 (0.14, 1.48) | 0.19 | 0.82 (0.20, 3.33) | 0.78 |

Note. * indicates statistical significance (*P* < 0.01). aOR = adjusted odds ratio. CI = confidence interval. Covariates include age, race, marital status, and NHANES survey cycle.
